# PI-MMNet: a cross-modal neural network for predicting neurological deterioration in pontine infarction

**DOI:** 10.3389/fnins.2025.1637079

**Published:** 2025-10-01

**Authors:** Hui Jin, Xiaona Xu, Yichan Ye, Xuhao Shan, Cheng Yang, Enyu Bao, Min Li, Weili Chen, Xuerong Huang, Jikui Liu, Hao Kou, Ruyue Huang

**Affiliations:** ^1^School of Computer Science, Hangzhou Dianzi University, Hangzhou, China; ^2^Department of Neurology, The Third Affiliated Hospital of Wenzhou Medical University, Ruian, China; ^3^HDU-ITMO Joint Institute, Hangzhou Dianzi University, Hangzhou, China; ^4^Institute of Intelligence Science and Engineering, Shenzhen Polytechnic University, Shenzhen, China; ^5^Shijiazhuang Posts and Telecommunications Technical College, Shijiazhuang, China

**Keywords:** neurological deterioration, pontine infarction, adaptive graph, dynamic fusion, multiple modality

## Abstract

**Introduction:**

Pontine infarction, a subtype of ischemic stroke, often leads to neurological deterioration (ND). Current diagnostic methods rely mainly on imaging and neglect clinical data, while existing multimodal models struggle with small lesions, heterogeneous inputs, and high computational cost.

**Methods:**

We propose PI-MMNet, a cross-modal neural network combining: (i) a Multi-modal Feature Processing module with Mamba-based extractors, (ii) a Dynamic Residual Fusion module for robust feature integration, and (iii) an Adaptive Graph module for efficient relational reasoning. A multi-loss strategy jointly optimizes alignment, graph consistency, and classification. Experiments used 386 pontine infarction cases with MRI and clinical data under 5-fold cross-validation.

**Results:**

PI-MMNet outperformed state-of-the-art methods, improving accuracy by 1.03%, F1 by 0.0504, and AUC by 0.0343, while using only $\frac{1}{46}$ parameters and $\frac{1}{35}$ memory of the strongest baseline. Ablation and visualization confirmed the contributions of all modules.

**Discussion:**

PI-MMNet provides an efficient and interpretable framework for predicting ND in pontine infarction and may generalize to other multimodal medical tasks. Our code is available at https://github.com/jinhui66/PI-MMNet.

## 1 Introduction

Stroke remains a global health priority, ranking as the second leading cause of death worldwide (Feigin et al., 2022) and a major contributor to long-term functional disability (Shimmyo and Obayashi, 2024). Ischemic stroke, the most prevalent subtype, accounts for substantial morbidity and mortality (Pohl et al., 2021). Pontine infarctions represent roughly 7% of all ischemic strokes. Within this group, some patients may experience neurological deterioration (ND) early in the disease course, typically within 48 to 72 h. This deterioration is marked by increasing motor weakness, worsening dysarthria, sensory deficits, or reduced consciousness (Wang et al., 2022; Knopman et al., 2009; Van Zandvoort et al., 2003; Huang et al., 2016; Yang H. et al., 2023). Timely identification of these high-risk patients is crucial for informed clinical decision-making and optimized patient care.

Traditional machine learning approaches, including logistic regression (Nusinovici et al., 2020) and ensemble methods (Yang et al., 2020; Seto et al., 2022), have demonstrated limited efficacy in leveraging imaging biomarkers due to their reliance on structured clinical data. In contrast, deep learning architectures have revolutionized feature extraction in domains from natural language processing (Li et al., 2024) to computer vision (He et al., 2016; Dosovitskiy et al., 2020; Yang J. et al., 2023), with extensions to medical imaging (Wu et al., 2024; Lu et al., 2023; Yang et al., 2025; Chen et al., 2023; Wang et al., 2024). However, conventional vision models like ResNet (He et al., 2016), Vision Transformer (Xin et al., 2024) and nnMamba (Gong et al., 2025) show suboptimal performance in detecting subtle pontine lesions, while unimodal approaches neglect complementary clinical data essential for comprehensive assessment, as evidenced by their inferior performance compared to other multimodal methods. Recent advances in prognostic modeling have been propelled by breakthroughs in multi-modal fusion techniques. In the management of intracerebral hemorrhage, DL-base (Pérez del Barrio et al., 2023) introduced a novel framework that seamlessly integrates radiomic features from admission CT scans with longitudinal clinical time-series data. Similarly, GCS-Net (Shan et al., 2023) demonstrated significant progress by fusing quantitative CT imaging metrics with Glasgow Coma Scale assessments to accurately predict patient outcomes. Together, these approaches highlight meaningful advancements in the development of more precise and effective prognostic models. General medical diagnostic systems (Zheng Shuai et al., 2022; Zhou et al., 2023; Yang et al., 2024; Xiao et al., 2025) further highlight the benefits of cross-modal integration. Despite recent advancements, existing fusion approaches often fall short in achieving complete integration of multi-modal features. For instance, methods like IRENE (Zhou et al., 2023) have introduced cross-modal attention mechanisms, yet they suffer from high computational costs and adopt attention and transformer based processing for fused features, having high complexity when dealing with long sequence features. Similarly, MOME (Xiong et al., 2024) has sought to improve feature extraction using a network of multi-modal experts, but these solutions bring new challenges, including high computational demands and large parameter counts. Additionally, current models struggle with performing comprehensive hierarchical processing of fused multi-modal representations, which could be revealed by they fuse the clinical and imaging data at the same level. But in real situations, there is a difference in the feature dimensions between clinical data and imaging data, and disease diagnosis may focus more on a single data. While significant progress has been made in the field of multi-modal fusion, there remains substantial room for improvement in enhancing both the depth of feature integration and the efficiency of these models.

Recent advancements in multi-modal learning have increasingly incorporated graph neural networks (GNNs) (Scarselli et al., 2008) for visual representation learning (Yan et al., 2018; Wang et al., 2019). Building on this trend, MMGL (Zheng Shuai et al., 2022) introduced a graph-based medical prediction framework that leverages graph convolutions to model fused multi-modal features, demonstrating strong discriminative power in distinguishing positive and negative samples on NC vs. ASD task and so on. Despite its success, the model encounters significant challenges when applied to predicting ND in pontine infarction cases. Factors such as varying lesion sizes, heterogeneous imaging patterns, and limited feature extraction depth constrain its predictive performance. These limitations underscore the need for more advanced graph-based approaches that can adaptively handle complex medical imaging data.

Existing medical diagnostic frameworks, while successful in addressing other neurological conditions (Yu et al., 2024; Ji et al., 2025), face notable challenges when applied to pontine infarction due to its unique pathological characteristics. Current methods often attempt to enhance performance by increasing model complexity through additional parameters. However, this strategy is inefficient for analyzing pontine infarction, where lesion variability and subtle clinical symptoms demand more advanced feature learning rather than simply larger models. Building on the limitations identified in previous multi-modal fusion and graph-based approaches, our goal is to develop an efficient and lightweight network architecture specifically optimized for pontine infarction analysis. Instead of relying on extensive parameter scaling, our methodology emphasizes intelligent feature extraction and fusion. This approach aims to provide clinicians with reliable decision support for diagnosing neurological deterioration, effectively addressing both the computational inefficiencies and performance deficiencies of existing solutions. In summary, our study focuses on developing a streamlined and efficient multi-modal network specifically for pontine infarction, aiming to accurately forecast early neurological deterioration. Current methods often depend on extensive parameter scaling, but our strategy prioritizes smart feature extraction, adaptive fusion, and graph-based modeling to tackle the specific challenges posed by pontine lesions. By adopting this approach, we aspire to not only drive methodological advancements in multi-modal learning but also offer clinically relevant decision support. This can enhance patient outcomes and optimize the allocation of healthcare resources.

To tackle the following challenges: (i) the presence of small and isolated lesion areas that often lead traditional CNN or Transformer models to miss subtle pathological signs; (ii) diverse imaging and clinical patterns that reduce the effectiveness of basic concatenation or shallow fusion methods; and (iii) computational inefficiencies in current multi-modal fusion and graph-based techniques, which typically enhance performance by scaling parameters rather than developing more meaningful representations. We introduce a multi-modal multi-loss network (PI-MMNet) designed to predict neurological deterioration in pontine infarction. Our key contributions include the following:

(1) To effectively identify subtle lesions, we have integrated Mamba-based feature extractors into the Multi-modal Feature Processing module. This approach allows for deeper and more discriminative representation learning compared with shallow CNN/FC encoders.

(2) To manage the challenges posed by heterogeneous modalities and inadequate fusion, we have developed the Dynamic Residual Fusion (DRF) module. This module employs cross-concatenation, residual preservation, and adaptive weighting, facilitating robust interactions and balanced contributions from both imaging and clinical features.

(3) For improving computational efficiency and enhancing relational reasoning, we have introduced an Adaptive Graph (AG) module. This module models inter-sample relationships using lightweight graph convolutions, which avoid excessive parameter scaling.

(4) To address the challenges of supervising cross-modal alignment and graph consistency, we propose a multi-loss learning strategy. This strategy integrates CSDM, GE, and CE losses, ensuring that feature integration, structural modeling, and classification are jointly optimized.

## 2 Materials and methods

### 2.1 Materials

#### 2.1.1 Dataset

**Figure 1:**
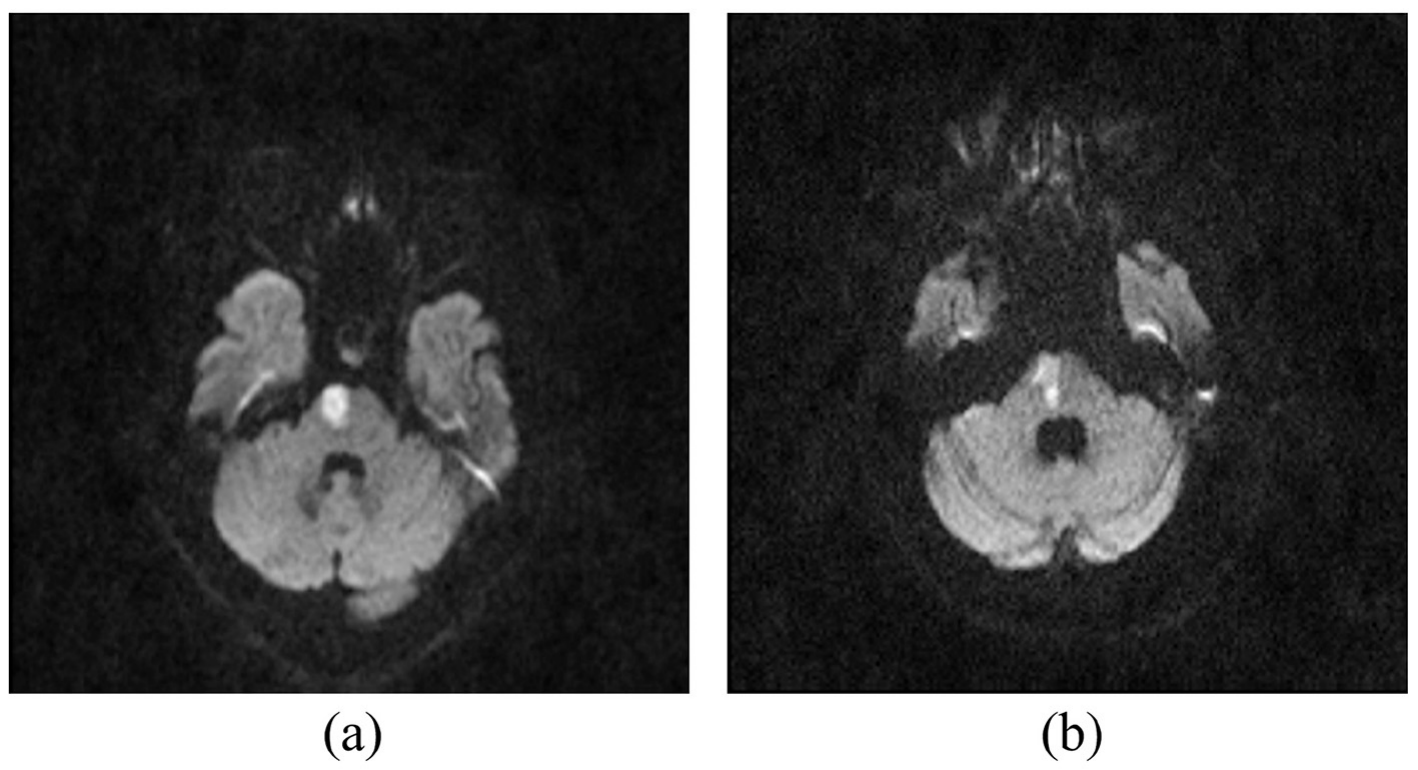
The MRI slices from the dataset, where **(a)** shows a sample slice from an ND case, and **(b)** presents a slice from a non-ND sample.

The study utilized a proprietary dataset comprising 386 pontine infarction cases obtained through a hospital partnership. Each case included raw magnetic resonance imaging (MRI) volumetric scans and corresponding clinical records, such as admission/discharge National Institutes of Health Stroke Scale (NIHSS) scores, length of hospital stay (LOHS), thrombolysis, vertebrobasilar artery stenosis (VAS), and other treatment-related parameters. MRI was performed using 1.5-T superconducting magnets (Magnet Avanto 1.5; Siemens, Erlangen, Germany). DWI scans [time of repetition (TR): 3,200 ms/time of echo (TE): 90 ms] were obtained at a 5-mm slice thickness. Figure 1 illustrates representative MRI slices with ground truth annotations, while Table 1 details the cohort's demographic distribution, functional assessment metrics, and clinical outcomes.

**Table 1:** This table summarizes the data distribution within our dataset.

**Feature**	**Positive case**	**Negative case**	**Correlation**
Gender	77/49 (male/female)	160/100 (male/female)	0.0041
Age	69.92 ± 10.96	69.96 ± 48.16	0.0004
LOHS	16.40 ± 8.11	13.05 ± 6.71	0.2148
Thrombolysis	14/112 (1/0)	28/232 (1/0)	0.0051
Admission NIHSS (1)	5.02 ± 2.23	4.91 ± 3.11	0.0193
Discharge NIHSS (2)	6.75 ± 2.48	3.93 ± 2.58	0.4618
(2) – (1)	1.72 ± 2.39	-0.98 ± 1.66	0.5487
VAS	31/95 (1/0)	30/229 (1/0)	0.1673
ND	126	260	-

For discrete variables, *n*_1_/*n*_2_ (A/B) represents that the number of A is *n*_1_ and the number of B is *n*_2_; for relatively continuous variables, it is represented by Avg ± Std. Correlation represents the correlation between the attribute and ND, with a value of [0,1]. Higher values indicate a stronger association between the attribute and ND.

The prediction task was defined as a binary classification of ND, with positive cases indicating progressive clinical worsening and negative cases representing stable or improved outcomes. To quantify the clinical progression of the patients' neurological symptoms, we measured their NIHSS score at the time of admission, at the time of maximal neurological deficit, and at the time of discharge. ND was defined as any ≥ 2-point increase in the total NIHSS score (not NIHSS at discharge) between the maximal and initial neurological deficits (Oh et al., 2012; Zong et al., 2022; Bao et al., 2023). Volumetric MRI data underwent standardized preprocessing, including skull stripping and intensity normalization, maintaining consistent spatial resolutions of 20 × 256 × 256 voxels. The model's tabular inputs are refined by evaluating their correlation with relevant factors. As shown in Table 1, the selected variables for the model include LOHS, NIHSS scores at admission and discharge, and the VAS. The model's final output provides a probabilistic prediction of ND progression based on integrated imaging and clinical features.

#### 2.1.2 Implementation details

Our experiments were performed on a system equipped with an NVIDIA 3090Ti GPU, using PyTorch version 2.5.0 and CUDA 11.8. To train the model, we implemented a multi-loss strategy alongside the Adam optimizer. This training process spanned 100 epochs and utilized specific hyperparameters: the initial learning rate was set at 1 × 10^−4^, the weight decay at 1 × 10^−7^, and the beta values at (0.9, 0.98). During validation, we selected the best models based on a balance of Accuracy (Acc), Recall, and Precision (Prec) metrics. These chosen models were then used to assess the performance on the test set.

For the purpose of data partitioning, we allocated 20% of the entire dataset solely for testing. The remaining 80% was employed in a 5-fold cross-validation process, with each cycle utilizing 64% of the data for training and 16% for validation. This approach resulted in five unique models, each independently evaluated using the designated test set. The overall performance was determined by statistically aggregating the evaluation results from these models, calculating both the mean values and standard deviations to thoroughly assess the models' effectiveness.

### 2.2 Methods

**Figure 2:**
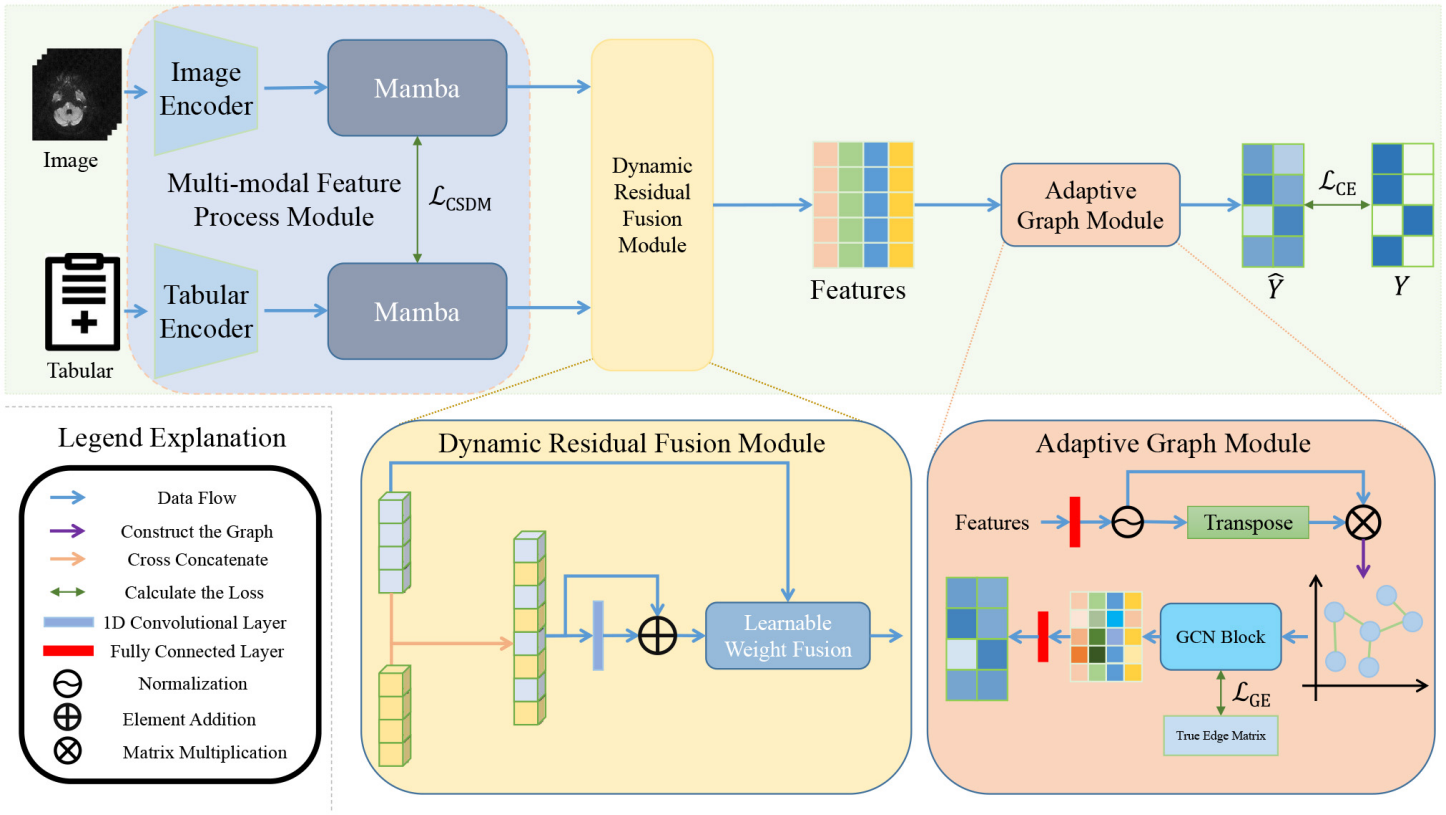
Overall framework of the proposed method. The legend explanation is located in the bottom left corner. The image data and clinical information are initially processed by the MFP module for feature extraction. These features are then integrated through the DRF module, culminating in the AG module generating the final classification output.

Our method utilizes a dual-branch architecture to separately process image and tabular data, as depicted in Figure 2. The pipeline is systematically structured into three distinct stages to ensure comprehensive data analysis.

The first stage involves the Multi-modal Feature Processing (MFP) module, where the image, denoted as *I*, is processed by an Image Encoder to extract deep visual features. Concurrently, tabular data, denoted as *T*, undergoes transformation via a dedicated Tabular Encoder. To enhance the expressive power of the unimodal features, Mamba blocks are introduced to refine both modalities, resulting in the image feature representation and tabular feature representation.

In the subsequent stage, we introduce the DRF module, which facilitates effective integration of features through three main strategies. First, cross-concatenation is employed to create a joint feature space. Second, residual connections help maintain the integrity of original features. Finally, adaptive weight adjustment dynamically balances the contributions of each modality. The outcome is a fused feature map *m*, enriched with cross-modal information.

The final stage involves graph structure modeling, where we develop an adaptive GNN. In the AG module, fused features from each sample are treated as graph nodes, with edges determined by binarized cosine similarity scores between samples, thresholded to ensure sparsity. A multi-layer graph convolutional network (GCN) performs message passing, enabling each node to aggregate information from its neighbors, thereby generating more discriminative graph-enhanced features. A lightweight classification head then processes these refined node representations to produce final sample-level predictions.

The entire framework is trained end-to-end, facilitating deep collaboration and complementary feature learning across modalities.

To assess the effectiveness of our approach, we conducted a detailed comparison with several leading models, including GBDT, ResNet-50, ViT, nnMamba, DL-base, MMGL, IRENE, and MOME (Seto et al., 2022; Hara et al., 2018; Dosovitskiy et al., 2020; Gong et al., 2025; Pérez del Barrio et al., 2023; Zheng Shuai et al., 2022; Zhou et al., 2023; Xiong et al., 2024). To ensure a fair evaluation, we carefully fine-tuned the default hyperparameters in all publicly available code to optimize performance across each model.

#### 2.2.1 Multi-modal feature processing module

We utilize several convolutional neural network (CNN) layers as the Image Encoder (*En*_*i*_) and several fully connected (FC) layers as the Tabular Encoder (*En*_*t*_). To enhance feature extraction, we implement the Mamba module (Gu and Dao, 2023) for extracting secondary features. In contrast to MLPs, RNNs, or Transformers, Mamba facilitates deep and discriminative representation learning while maintaining a lower computational cost, making it particularly suitable for handling diverse medical data. The features are extracted using the following equations:


(1) $$\begin{array}{l} f_{\theta }\left(x\right)=M_{\theta }\left(En_{\theta }\left(x\right)\right), \end{array}$$

where $M_{\theta }$ and *En*_θ_ denote Mamba operation and Encoder operation, respectively, acting on input *x* of modality θ ∈ {*i, t*}.

#### 2.2.2 Dynamic residual fusion module

In the DRF module, we draw inspiration from ITCFN (Hu et al., 2025) to construct cross-modality features by interleaving the input features using a Cross Concat operation:


(2) $$\begin{array}{l} \text{O}=Cross\;Concat\left(f_{i}\left(I\right),f_{t}\left(T\right)\right), \end{array}$$

where the even-indexed channels of **O** are filled with *f*_*i*_(*I*) (*i*.*e*., **O**[0::2] = *f*_*i*_(*I*)), while the odd-indexed channels are filled with *f*_*t*_(*T*) (*i*.*e*., **O**[1::2] = *f*_*t*_(*T*)). This interleaving technique ensures a dense interaction between the two modalities, maintaining their distinct representations.

Next, we process **O** through a CNN layer and concatenate it with itself. Drawing from CSF-NET (Shen et al., 2025), we compute the fused feature map *m* as a weighted combination of this concatenated result and the input image feature:


(3) $$\begin{array}{l} m=\omega_{1}\cdot \left(Conv\left(\text{O}\right)+\text{O}\right)+\omega_{2}\cdot f_{i}\left(I\right),\;where\;\omega_{1}+\omega_{2}=1, \end{array}$$

where *Conv* denotes the 1D convolution operation, applied to the interleaved features. The weights ω_1_ and ω_2_ are dynamically adjustable, allowing the model to adapt to various types of data.

#### 2.2.3 Adaptive graph module

Inspired by MMGL (Zheng Shuai et al., 2022), the input features *m* ∈ ℝ^*N*×*Length*^ represent *N* nodes, each defined by a feature vector of dimension *Length*. Initially, these features undergo transformation via a fully connected layer, followed by normalization to generate N. This normalized output is calculated as:


(4) $$\begin{array}{l} N=Norm\left(\alpha_{1}\cdot m+\beta_{1}\right), \end{array}$$

where α_1_ and β_1_ are trainable parameters optimized during training, and *Norm* denotes the feature normalization process. To establish relationships between nodes, an affinity matrix is constructed by multiplying the normalized features with their transpose:


(5) $$\begin{array}{l} W=N\times N^{⊤}, \end{array}$$

where × signifies matrix multiplication, resulting in a matrix *W* ∈ ℝ^*N*×*N*^, where each element indicates the similarity between node pairs. A thresholding operation at 0.95 converts the affinity matrix into binary connections:


(6) $$A_{ij}=\{\begin{array}{ll} 1, & \text{if }W_{ij}\geq 0.95,\\ 0, & \text{if }W_{ij}<0.95. \end{array}$$

The binary edge matrix is reformatted into an edge index list *E* ∈ ℝ^*E*×2^, where *E* is the total number of edges. Each entry *E*_*k*_ = [*i, j*] represents a connection between nodes *i* and *j*, as determined during thresholding.

The final output ŷ are computed using a *GCN*, refined by additional trainable parameters:


(7) $$\begin{array}{l} ŷ=Softmax\left(\alpha_{2}\cdot GCN\left(m,E\right)+\beta_{2}\right), \end{array}$$

where α_2_ and β_2_ further enhance the graph-convolved features.

#### 2.2.4 Loss function

Our loss function is designed to optimize model performance by incorporating three essential components: the cosine similarity distribution matching (CSDM) loss, the graph edge (GE) loss, and the cross-entropy (CE) loss.

##### 2.2.4.1 CSDM loss

Drawing inspiration from (Jiang and Ye 2023), we employ the CSDM loss to align cross-modal feature distributions through Kullback-Leibler (KL) divergence minimization between predicted and ground-truth cosine similarity distributions. For a batzh of image features *f*^*I*^ ∈ ℝ^*N*×*Length*^ and tabular features *f*^*T*^ ∈ ℝ^*N*×*Length*^, we compute:


(8) $$\begin{array}{l} L_{CSDM}=\frac{1}{2N}\overset{N}{\underset{i=1}{\sum }}\left[D_{KL}\left(p_{i}^{i2t}||q_{i}^{i2t}\right)+D_{KL}\left(p_{i}^{t2i}||q_{i}^{t2i}\right)\right], \end{array}$$

where $p_{i}^{i2t},p_{i}^{t2i}$ represent the predicted similarity distributions from image to tabular data and from tabular to image data, respectively. The corresponding ground-truth one-hot distributions are denoted by $q_{i}^{i2t},q_{i}^{t2i}$.

##### 2.2.4.2 GE loss

The GE loss serves as a regularization term that enforces structural consistency between the predicted adjacency matrix *A* and the ground-truth adjacency matrix A^*^. Formally, for a graph with *N* nodes, the true adjacency matrix A^*^ ∈ ℝ^*N*×*N*^ is constructed based on node labels. The matrix is defined as follows:


(9) $$\{\begin{array}{ll} 1, & \text{if }y_{i}=y_{j},\\ 0, & \text{if }y_{i}\neq y_{j}, \end{array}$$

where *y*_*i*_ denotes the ground-truth class of the *ith* sample.

The GE loss is then defined as the L2-norm distance between the predicted and ground-truth adjacency matrices:


(10) $$\begin{array}{l} L_{GE}=||A-A^{*}{||}_{2}, \end{array}$$

where ||·||_2_ denotes the Frobenius norm (also known as the L2 matrix norm), which measures the element-wise Euclidean distance between the two matrices. This loss term encourages the predicted graph structure to align with the semantic relationships implied by the node labels.

##### 2.2.4.3 CE loss

The standard CE loss supervises the classification task:


(11) $$\begin{array}{l} L_{CE}=-\frac{1}{N}\overset{N}{\underset{i=1}{\sum }}y_{i}log\left({ŷ}_{i}\right), \end{array}$$

where *y*_*i*_ and ŷ_*i*_ denote ground-truth and predicted class probabilities, respectively.

The total loss function is expressed as a weighted sum of three components:


(12) $$\begin{array}{l} L_{total}={\text{λ}}_{1}L_{CSDM}+{\text{λ}}_{2}L_{GE}+{\text{λ}}_{3}L_{CE}, \end{array}$$

where the coefficients λ_1_, λ_2_ and λ_3_ determine the relative importance of each component in the overall loss calculation. In our implementation, these weights are assigned values of 0.2, 0.4, and 0.4 for λ_1_, λ_2_ and λ_3_, respectively.

## 3 Results

### 3.1 Comparative experiments

We assessed performance using several metrics: Acc, Recall, Prec, F1 score, Area Under the Curve (AUC), model size (Params), and training memory consumption (Memory). As detailed in Table 2, our method consistently surpassed all baseline models in predictive performance, improving Acc, Recall, Prec, F1 score, and AUC by at least 1.03%, 5.21%, 0.55%, 0.0504, and 0.0243, respectively.

**Table 2:** Comparative experiments between our method and other methods.

**Method**	**Modality**	**Acc (%)**	**Recall (%)**	**Prec (%)**	**F1 (10^−2^)**	**AUC (10^−2^)**	**Params**	**Memory**
GBDT (Seto et al., 2022)	T	61.30	48.33	32.60	36.78	50.37	-	-
		[46.03–76.57]	[18.34–79.32]	[15.61–49.59]	[16.16–57.40]	[19.91–80.83]		
ResNet-50 (Hara et al., 2018)	I	59.32	44.55	34.96	37.77	54.29	4,615K	10G
		[57.07–61.57]	[20.62–68.48]	[30.79–39.13]	[28.15–47.39]	[51.04–57.54]		
ViT (Dosovitskiy et al., 2020)	I	66.08	43.64	49.92	44.79	64.30	8,712K	12G
		[63.46–68.70]	[33.25–54.03]	[31.98–67.86]	[35.53–54.05]	[50.83–77.77]		
nnMamba (Gong et al., 2025)	I	51.43	61.60	37.28	43.13	56.48	1,287K	13G
		[38.55–64.31]	[30.03–93.17]	[33.45–41.11]	[35.76–50.50]	[50.75–62.21]		
DL-base (Pérez del Barrio et al., 2023)	I+T	80.78	58.10	69.83	62.44	79.97	30K	6,248M
		[75.80–85.76]	[49.58–66.62]	[54.48–85.18]	[55.08–69.80]	[78.40–81.54]		
MMGL (Zheng Shuai et al., 2022)	I+T	81.30	50.37	80.68	61.78	74.61	13K	5,926M
		[80.14–82.46]	[47.06–53.68]	[74.19–87.17]	[60.78–62.78]	[69.98–79.24]		
IRENE (Zhou et al., 2023)	I+T	72.73	55.32	50.92	52.00	69.47	5,848K	18G
		[68.83–76.63]	[34.57–76.07]	[41.84–60.00]	[38.36–65.64]	[63.77–75.17]		
MOME (Xiong et al., 2024)	I+T	84.42	69.57	77.85	72.52	86.56	1,933K	21G
		[83.12–85.72]	[59.37–79.77]	[69.15–86.55]	[69.57–75.47]	[82.80–90.32]		
Ours	I+T	85.45	74.78	81.23	77.56	88.99	40K	5,932M
		[84.87–86.03]	[67.64–81.92]	[73.79–88.67]	[72.83–82.29]	[88.06–89.92]		

I stands for image modality, and T stands for tablular modality. The best and second-best results are in red and blue, respectively.

Regarding efficiency, our model ranked third in terms of Params and second in Memory usage, demonstrating a favorable balance between performance and resource utilization. While MMGL showed lower computational demands, it did so at the expense of significantly reduced Acc, underscoring our method's ability to achieve superior predictive power while maintaining competitive efficiency. Our approach significantly outperforms MOME, the leading model among all others, by increasing Acc by 1.03%, the F1 score by 0.0504, and the AUC by 0.0343. Moreover, it achieves these improvements while utilizing only approximately $\frac{1}{46}$ of the parameters and $\frac{1}{35}$ of the memory required by MOME. In summary, our method effectively balances enhanced performance with reduced model complexity, achieving state-of-the-art results with exceptional computational efficiency.

### 3.2 Ablation experiments

#### 3.2.1 Ablation experiments based on modules

Table 3 presents an ablation study designed to investigate the contributions of three critical components in our model: the MFP module, DRF module, and AG module. To assess their impact, we conducted experiments by (1) replacing the Mamba blocks in the MFP module with multiple CNN and FC layers, (2) substituting the DRF module with direct concatenation of cross-modal features, and (3) replacing the AG module with FC layers for output generation. This systematic analysis quantifies the significance of each module.

**Table 3:** Ablation experiments on the modules.

**MFP**	**DRF**	**AG**	**Acc (%)**	**Recall (%)**	**Prec (%)**	**F1 (10^−2^)**	**AUC (10^−2^)**
✓	-	-	79.22	54.61	69.09	60.34	77.35
			[73.63-84.81]	[39.57–69.65]	[56.54–81.64]	[48.30–72.38]	[71.81–82.89]
-	✓	-	82.60	62.61	76.10	67.88	79.21
			[78.86–86.34]	[50.08–75.14]	[65.59–86.61]	[59.34–76.42]	[74.73–83.69]
-	-	✓	82.08	57.22	77.60	65.66	77.96
			[76.61–87.55]	[51.18–63.26]	[62.46–92.74]	[56.43–74.89]	[74.63–81.29 ]
✓	✓	-	84.68	66.09	80.50	72.09	84.20
			[82.98–86.38]	[61.32–70.86]	[71.10–89.90]	[70.97–73.21]	[80.29–88.11]
✓	-	✓	83.64	60.87	79.22	68.37	81.12
			[78.73–88.55]	51.65–70.09]	[66.16–92.28]	[60.06–76.68]	[75.94–86.30]
-	✓	✓	83.12	60.00	80.94	67.54	88.26
			[81.82–84.42]	[46.81–73.19]	[72.04–89.84]	[63.23–71.85]	[86.78–89.74]
✓	✓	✓	85.45	74.78	81.23	77.56	88.99
			[84.87–86.03]	[67.64–81.92]	[73.79–88.67]	[72.83–82.29]	[88.06–89.92]

The best and second-best results are in red and blue, respectively.

Incorporating the MFP module results in substantial enhancements to performance metrics, with accuracy rising by 1.56% to 2.33% and the F1 score improving by 0.0421 to 0.1002, in comparison to configurations without this module. These advancements are primarily due to the shallow CNN and FC layers' inadequacies in processing intricate medical image feature extraction. In contrast, the deep feature extractor based on Mamba offers a more solid foundation for subsequent feature fusion and classification tasks.

The significance of the DRF module is clearly demonstrated in its ablation results, where its removal leads to substantial declines in performance. Accuracy decreases by 1.04% to 5.46%, and the F1 score falls by 0.0188 to 0.1175. These findings underscore the DRF module's essential role in achieving effective multimodal feature fusion. Our approach integrates two principal strategies: cross-concatenation and weighted combination. During subsequent 1D convolution operations, cross-concatenation surpasses direct-concatenation by merging bimodal features at the same level through 1D convolution. Meanwhile, the weighted combination mechanism allows for adaptive adjustment of bimodal feature weights, collectively enhancing the learning of complex fused representations.

The importance of the AG module is highlighted in ablation studies, where its removal leads to a marked reduction in model performance. An accuracy improvement between 0.52% and 4.42% emphasizes the AG module's critical role in the overall architecture. Utilizing graph convolution techniques, the module enhances feature similarity among nodes and increases the differentiation between dissimilar features. This design exhibits greater proficiency in distinguishing between positive and negative samples compared to traditional convolution methods, thereby facilitating more precise ND prediction.

Model performance demonstrably improves with the reintroduction of each previously omitted module. When all three modules are integrated into the full PI-MMNet framework, the model reaches peak performance. This result highlights the effectiveness and essential role of each component within the system. In our comprehensive approach, the MFP module extracts deep-level features from the input data, the DRF module efficiently fuses bimodal features, and the AG module utilizes graph network structures to accurately classify samples. This thoughtfully constructed modular architecture ensures optimal performance through the synergistic interaction of all components.

#### 3.2.2 Ablation experiments based on modalities

To assess the impact of each modality, we conducted ablation experiments focusing on individual modalities. The results, detailed in Table 4, clearly demonstrate that combining modalities significantly enhances performance compared to using a single modality, whether Image or Tabular. Specifically, when comparing bimodal results with those of single modalities on our dataset, we observe improvements in accuracy, recall, precision, F1 score, and AUC by 4.22%, 9.56%, 7.63%, 0.0904, and 0.0573, respectively. These findings confirm that multimodal learning effectively utilizes cross-modal information to boost model performance.

**Table 4:** Ablation experiments on the modalities.

**Modality**	**Acc (%)**	**Recall (%)**	**Prec (%)**	**F1 (10^−2^)**	**AUC (10^−2^)**
Image	73.15	50.73	66.89	57.48	64.28
	[68.95–77.35]	[43.86–57.60]	[60.71–73.07]	[51.86–63.10]	[56.94–71.62]
Tabular	81.23	65.22	73.60	68.52	83.26
	[73.26–89.20]	[53.71–76.73]	[69.94–77.26]	[63.51–73.53]	[76.54–89.98]
Ours (Image + Table)	85.45	74.78	81.23	77.56	88.99
	[84.87–86.03]	[67.64–81.92]	[73.79–88.67]	[72.83–82.29]	[88.06–89.92]

The best and second-best results are in red and blue, respectively.

#### 3.2.3 Ablation experiments based on loss function

The ablation studies summarized in Table 5 highlight important insights into the effectiveness of various loss term combinations. Notably, incorporating any two loss terms consistently outperformed using the CE loss alone. Specifically, adding either the CSDM or GE loss to the CE loss resulted in improvements of at least 0.78% in Acc, 0.86% in Recall, 2.81% in Prec, 0.0368 in F1 score, and 0.0192 in AUC. Furthermore, our proposed three-term loss function demonstrated superior performance across all metrics compared to any dual-term loss combinations. The improvements ranged from 1.55% to 1.29% in Acc, 4.35% to 1.75% in Recall, and 5.46% to 3.77% in Prec. Other key metrics also showed enhancements, with F1 score increasing by 0.0550 to 0.0286 and AUC gaining 0.0364 to 0.0214.

**Table 5:** The results of the ablation experiments based on the loss function, where CE loss is an indispensable loss component.

**CSDM**	**GE**	**CE**	**Acc (%)**	**Recall (%)**	**Prec (%)**	**F1(10^−2^)**	**AUC(10^−2^)**
-	-	✓	83.12	69.57	72.96	71.02	83.43
			[82.20–84.04]	[63.42–75.72]	[69.97–75.95]	[68.50–73.54]	[82.33–84.53]
✓	-	✓	83.90	70.43	75.77	72.06	85.35
			[82.42–85.38]	[59.18–81.68]	[68.16–83.38]	[68.44–75.68]	[79.77–90.93]
-	✓	✓	84.16	73.03	77.46	74.70	86.85
			[83.07–85.25]	[65.90–80.20]	[68.89–86.03]	[70.73–78.71]	[85.33–88.37]
✓	✓	✓	85.45	74.78	81.23	77.56	88.99
			[84.87–86.03]	[67.64–81.92]	[73.79–88.67]	[72.83–82.29]	[88.06–89.92]

The best and second-best results are in red and blue, respectively.

These experimental results confirm the individual contributions of each loss component and highlight their synergistic effects when combined. The consistent performance gains across multiple evaluation metrics underscore the complementary nature of the different loss terms within our multi-loss framework.

### 3.3 Analysis of hyperparameters in loss function

To validate the rationality of the hyperparameter selection, we conducted a comprehensive analysis of λ_1_, λ_2_, and λ_3_ combinations while maintaining their sum as 1. As shown in Table 6, the experimental results demonstrate that the optimal configuration (λ_1_ = 0.2, λ_2_ = 0.4, λ_3_ = 0.4) significantly outperforms the second-best combination (λ_1_ = 0.2, λ_2_ = 0.2, λ_3_ = 0.6) across all evaluation metrics. Specifically, it achieves superior performance with absolute improvements of 0.82% in Acc, 1.66% in Recall, 3.78% in Prec, 0.0244 in F1 score, and 0.0176 in AUC. These substantial gains confirm the robustness of our selected hyperparameter configuration and validate its effectiveness in optimizing model performance.

**Table 6:** Analysis of hyperparameters in loss function.

**λ_1_**	**λ_2_**	**λ_3_**	**Acc (%)**	**Recall (%)**	**Prec (%)**	**F1 (10^−2^)**	**AUC (10^−2^)**
0.2	0.2	0.6	84.63	73.12	77.45	75.12	87.23
			[83.91–85.35]	[68.23–78.01]	[72.56–82.34]	[72.45–77.79]	[86.34–88.12]
0.2	0.4	0.4	85.45	74.78	81.23	77.56	88.99
			[84.87–86.03]	[67.64–81.92]	[73.79–88.67]	[72.83–82.29]	[88.06–89.92]
0.2	0.6	0.2	84.37	72.86	76.89	74.67	86.92
			[83.65–85.09]	[67.45–78.27]	[71.34–82.44]	[71.89–77.45]	[85.89–87.95]
0.4	0.2	0.4	83.85	71.23	74.56	72.78	85.43
			[83.12–84.58]	[65.34–77.12]	[69.87–79.25]	[70.12–75.44]	[84.35–86.51]
0.4	0.4	0.2	83.97	70.89	75.12	72.89	85.78
			[83.25–84.69]	[65.12–76.66]	[70.23–80.01]	[70.34–75.44]	[84.67–86.89]
0.6	0.2	0.2	83.26	69.78	73.45	71.45	84.12
			[82.54–83.98]	[63.45–76.11]	[68.78–78.12]	[68.78–74.12]	[83.01–85.23]

The best and second-best results are in red and blue, respectively.

### 3.4 t-SNE visualization

**Figure 3:**
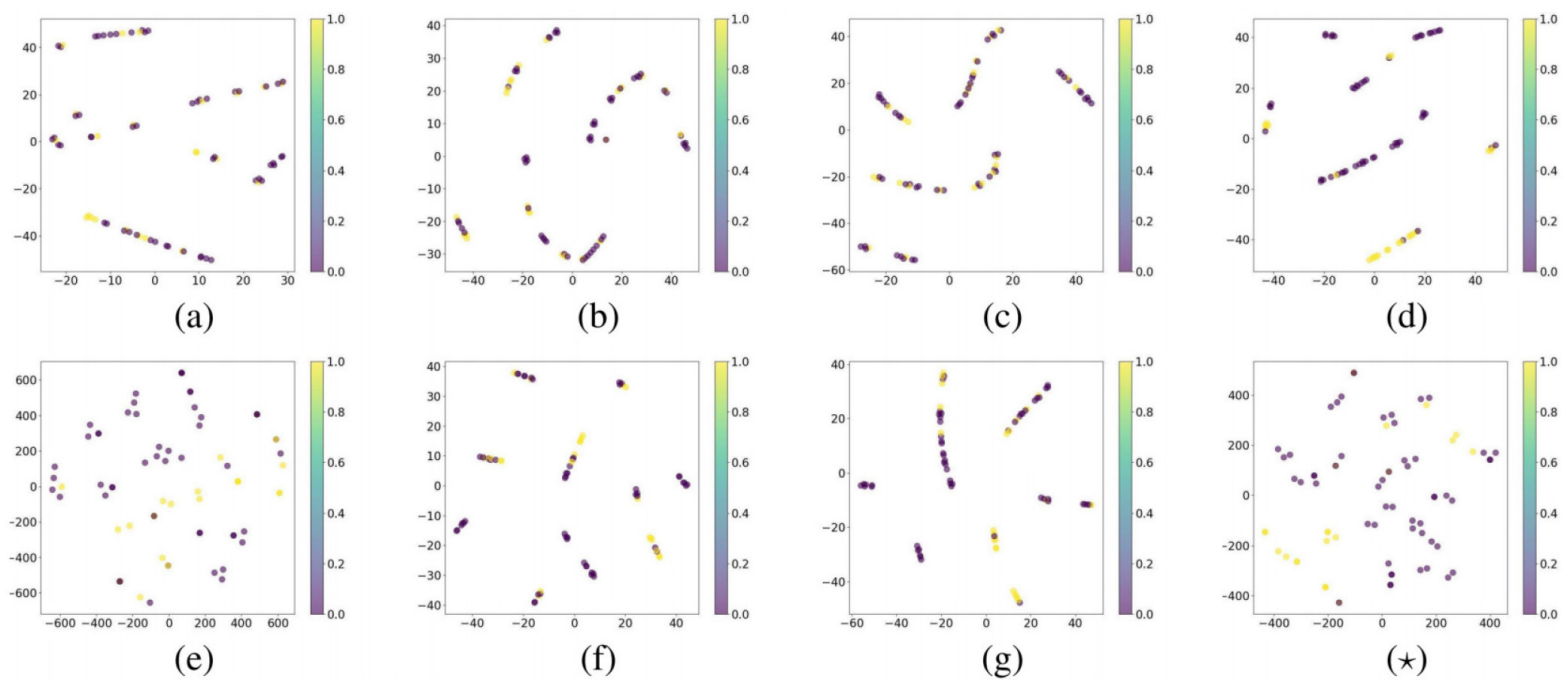
Visualize the features using t-SNE on the test set. **(a–g)** and **(⋆)** represent the results of employing ResNet-50, ViT, nnMamba, DL-base, MMGL, IRENE, MOME, and PI-MMNet.

To effectively visualize the features extracted by the network for each method, we employed the t-SNE technique (Van der Maaten and Hinton, 2008), which reduces feature dimensionality for clearer display. As shown in Figure 3, we applied both comparative and our visualization methods to the test set. In this visualization, the perplexity value is set to 3, where yellow points indicate positive labels and purple points represent negative labels.

The models ResNet, ViT, and nnMamba, represented in (a), (b), and (c) respectively, demonstrate some degree of clustering for single-modal inputs. However, many data points are tightly packed, making it challenging to clearly distinguish positive from negative samples. In contrast, the multi-modal input models DL-base, IRENE, and MOME, depicted in (d), (f), and (g), show improved differentiation between positive and negative samples. Nevertheless, these models display multiple continuous feature distributions with substantial overlap, limiting their effectiveness. The MMGL method, which utilizes graph networks and is illustrated in (e), achieves more discrete feature distributions. However, compared to PI-MMNet, indicated by (⋆), MMGL still lacks a clear boundary between most positive and negative samples, highlighting the superior separation capability of PI-MMNet.

Notably, t-SNE visualizations of graph-based approaches such as MMGL and PI-MMNet demonstrate distinct clustering patterns compared to conventional methods. This difference is due to the unique graph-structured representation learning paradigm these models employ. Unlike standard architectures that process samples independently, graph networks capture inter-sample relationships through advanced topological modeling and iterative message-passing mechanisms. This relational inductive bias significantly alters the geometry of feature distribution, arranging samples in latent space based on their intrinsic properties as well as their learned contextual relationships with other instances. As a result, the clusters formed exhibit greater discreteness and structural organization.

### 3.5 Grad-CAM visualization

**Figure 4:**
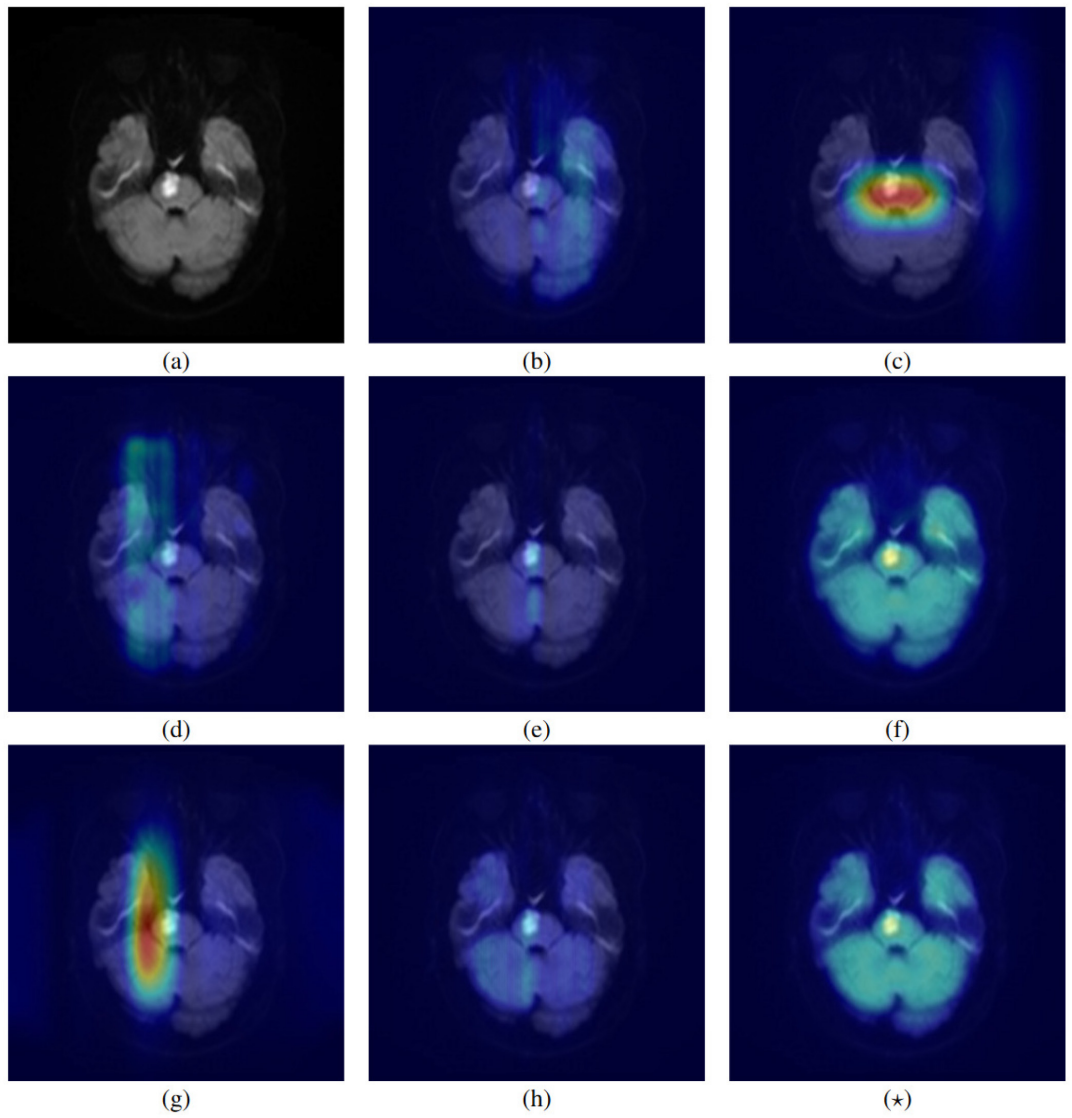
This figure provides an example showing the activation areas of various models for image input. **(a)** Represents the original image, **(b–h)** and **(⋆)** represent the result of ResNet-50, ViT, nnMamba, DL-base, MMGL, IRENE, MOME, and PI-MMNet, respectively. Among them, the more biased towards the red area, the higher the model's attention to the area.

To improve model interpretability, we utilized Grad-CAM (Selvaraju et al., 2017) to produce heatmap visualizations for various models. Figure 4 displays the visualization results of the original image along with different comparative methods. The unimodal models, ResNet, ViT, and nnMamba, shown in panels (b), (c), and (d), focus on predicting ND based solely on image data. These visualizations indicate that the models consistently apply medium-to-high attention weights to the lesion areas.

For multimodal approaches, including DL base, MMGL, IRENE, MOME, and PI MMNet, depicted in panels (e) through (h) and (⋆), ND prediction is conducted using both image and tabular data inputs. Within these models, DL base, IRENE, and MOME demonstrate moderate-to-high attention to the lesion area. Conversely, MMGL and PI-MMNet exhibit distinct attention patterns, moderately focusing on broader brain regions while maintaining high attention on the lesion area.

This comparative analysis underscores the impact of architectural design on model attention patterns. Multimodal methods tend to offer more nuanced feature localization than unimodal approaches, enhancing the precision of lesion detection.

## 4 Discussion

Our PI-MMNet framework offers an innovative and effective approach for predicting ND in patients with pontine infarction by integrating multi-modal imaging data and clinical records. Our model synergistically combines MRI data with critical clinical indicators, such as NIHSS scores, providing a comprehensive and nuanced assessment of patient status that aligns with recent advances in personalized medicine approaches (Pérez del Barrio et al., 2023). The experimental results demonstrate that PI-MMNet not only achieves state-of-the-art performance across multiple evaluation metrics but does so with significantly improved computational efficiency, utilizing only a fraction of the parameters and memory required by leading multimodal models such as MOME (Xiong et al., 2024), thereby addressing the critical need for efficient clinical decision support systems.

A key strength of PI-MMNet lies in its modular architecture, which is specifically designed to address the unique challenges posed by pontine infarction, including small lesion size, heterogeneous imaging patterns, and the need for efficient cross-modal fusion. The MFP module, enhanced with Mamba-based feature extractors (Gu and Dao, 2023), enables deeper and more discriminative representation learning compared to conventional CNN or FC encoders, building upon recent advances in state-space models for medical imaging. This is particularly critical for capturing subtle pathological signs that are often missed by standard models (Bao et al., 2023). The DRF module facilitates robust interaction between imaging and clinical features through cross-concatenation, residual connections, and adaptive weighting, effectively overcoming the limitations of naive concatenation or shallow fusion methods documented in previous studies (Hu et al., 2025; Shen et al., 2025). Furthermore, the AG module leverages lightweight graph convolutions to model inter-sample relationships, enhancing relational reasoning without excessive parameter scaling, a notable advantage over computationally expensive attention- or transformer-based fusion methods (Zhou et al., 2023; Dosovitskiy et al., 2020).

Our multi-loss optimization strategy, which integrates CSDM, GE, and CE losses, ensures effective cross-modal alignment, structural consistency, and classification robustness. This multi-objective approach aligns with recent trends in medical AI that emphasize the importance of comprehensive optimization frameworks. This approach not only improves performance on individual metrics but also enhances the model's generalization capability, as evidenced by the ablation studies, t-SNE visualizations and Grad-CAM visualization. The latter revealed that PI-MMNet learns more discriminative and well-separated feature representations compared to both unimodal and multimodal baselines, including graph-based models like MMGL (Zheng Shuai et al., 2022), demonstrating the effectiveness of our architectural innovations.

These advancements are particularly relevant in the context of current state-of-the-art methods, which often rely on heavy parameterization and lack specialized mechanisms for handling the intricacies of pontine infarction. For instance, while models such as IRENE (Zhou et al., 2023) and MOME have made strides in multimodal fusion, they remain computationally burdensome and less suited to tasks requiring fine-grained feature extraction from small or ambiguous lesions. In contrast, PI-MMNet offers a lightweight yet powerful alternative that balances performance with efficiency, a crucial consideration for clinical deployment where resources may be limited.

Beyond pontine infarction, the design principles underlying PI-MMNet, including selective state-space modeling, dynamic residual fusion, and adaptive graph reasoning, hold promise for other medical domains involving multimodal data integration, such as Alzheimer's disease diagnosis, tumor malignancy prediction, or outcome forecasting in other stroke subtypes. The ability to handle heterogeneous data types with limited parameters also suggests potential utility in resource-constrained settings or edge computing environments, addressing an important gap in global healthcare accessibility (Pohl et al., 2021).

In conclusion, PI-MMNet represents a meaningful step forward in the prediction of neurological deterioration in pontine infarction. By combining innovative architecture design with efficient multimodal learning, our framework not only addresses specific challenges in current literature but also provides a scalable and interpretable tool for clinical decision support, potentially contributing to improved patient outcomes and optimized healthcare resource allocation (Shimmyo and Obayashi, 2024).

## 5 Conclusion

We present PI-MMNet, an innovative framework designed for predicting neurological deficits in pontine infarction. This model integrates three key modules: the MFP module for multi-modal feature extraction, the DRF module for adaptive feature fusion, and the AG module for attention-based decoding. By combining imaging and clinical data under a multi-loss optimization strategy, our approach efficiently utilizes computational resources, as evidenced by its fewer parameters and reduced storage requirements. Despite these efficiencies, our method surpasses existing models in performance metrics. Ablation studies emphasize the importance of each component, demonstrating that every module and loss function is critical to the framework's success. Although PI-MMNet demonstrates significant advancements, it has certain limitations. These include its reliance on single-center data and the absence of explicit lesion segmentation, which could impact its generalizability and precision. Future research will aim to address these issues by validating the model across multiple centers and incorporating automated lesion localization. Additionally, efforts will be made to handle missing modality issues to enhance its clinical applicability.

## Data Availability

The data analyzed in this study is subject to the following licenses/restrictions: the study utilized a proprietary dataset comprising 386 pontine infarction cases obtained through a hospital partnership. Each case included raw MRI volumetric scans and corresponding clinical records, such as admission/discharge National Institutes of Health Stroke Scale scores, length of hospital stay, and other treatment-related parameters. Requests to access these datasets should be directed to Ruyue Huang, zbyhry@163.com.

## References

[B1] Bao J. Liu N. Zhang J. Cai M. Chao L. Liu D.. (2023). Clinical features and predictors of early neurological deterioration in acute isolated pontine infarction. Zhonghua yi xue za zhi 103, 32–37. 10.3760/cma.j.cn112137-20220421-00886 36594135

[B2] Chen Y. Liu C. Huang W. Cheng S. Arcucci R. Xiong Z. (2023). Generative text-guided 3d vision-language pretraining for unified medical image segmentation. arXiv preprint arXiv:2306.04811. 10.48550/arXiv.2306.04811

[B3] Dosovitskiy A. Beyer L. Kolesnikov A. Weissenborn D. Zhai X. Unterthiner T.. (2020). An image is worth 16 *times* 16 words: transformers for image recognition at scale. *arXiv preprint arXiv:2010.11929*. 10.48550/arXiv.2010.11929

[B4] Feigin V. L. Brainin M. Norrving B. Martins S. Sacco R. L. Hacke W.. (2022). World stroke organization (WSO): global stroke fact sheet 2022. Int. J. Stroke 17, 18–29. 10.1177/17474930211065917 34986727

[B5] Gong H. Kang L. Wang Y. Wang Y. Wan X. Wu X.. (2025). “Nnmamba: 3D biomedical image segmentation, classification and landmark detection with state space model,” in 2025 IEEE 22nd International Symposium on Biomedical Imaging (ISBI) (IEEE: Houston, TX, USA), 1–5. 10.1109/ISBI60581.2025.10980694

[B6] Gu A. Dao T. (2023). Mamba: linear-time sequence modeling with selective state spaces. arXiv preprint arXiv:2312.00752. 10.48550/arXiv.2312.00752

[B7] Hara K. Kataoka H. Satoh Y. (2018). “Can spatiotemporal 3D CNNS retrace the history of 2D CNNS and imagenet?,” in Proceedings of the IEEE Conference on Computer Vision and Pattern Recognition (CVPR) (Salt Lake City, UT: IEEE), 6546–6555. 10.1109/CVPR.2018.00685

[B8] He K. Zhang X. Ren S. Sun J. (2016). “Deep residual learning for image recognition,” in Proceedings of the IEEE Conference on Computer Vision and Pattern Recognition (CVPR) (Las Vegas, NV: IEEE), 770–778. 10.1109/CVPR.2016.90

[B9] Hu X. Shen X. Sun Y. Shan X. Min W. Su L.. (2025). ITCFN: incomplete triple-modal co-attention fusion network for mild cognitive impairment conversion prediction. arXiv preprint arXiv:2501.11276. 10.1109/ISBI60581.2025.10980706

[B10] Huang R. Zhang X. Chen W. Lin J. Chai Z. Yi X. (2016). Stroke subtypes and topographic locations associated with neurological deterioration in acute isolated pontine infarction. J. Stroke Cerebrovasc. Dis. 25, 206–213. 10.1016/j.jstrokecerebrovasdis.2015.09.019 26508683

[B11] Ji Y. Xiao X. Chen G. Xu H. Ma C. Zhu L.. (2025). Cibr: Cross-modal information bottleneck regularization for robust clip generalization. arXiv preprint arXiv:2503.24182. 10.1007/978-3-032-04558-4_20

[B12] Jiang D. Ye M. (2023). “Cross-modal implicit relation reasoning and aligning for text-to-image person retrieval,” in Proceedings of the IEEE/CVF Conference on Computer Vision and Pattern Recognition (CVPR) (Vancouver: IEEE), 2787–2797. 10.1109/CVPR52729.2023.00273

[B13] Knopman D. S. Roberts R. O. Geda Y. E. Boeve B. F. Pankratz V. S. Cha R. H.. (2009). Association of prior stroke with cognitive function and cognitive impairment: a population-based study. Arch. Neurol. 66, 614–619. 10.1001/archneurol.2009.30 19433661 PMC3050015

[B14] Li B. Sun B. Li S. Chen E. Liu H. Weng Y.. (2024). Distinct but correct: generating diversified and entity-revised medical response. Sci. China Inf. Sci. 67:132106. 10.1007/s11432-021-3534-9

[B15] Lu S. Liu Y. Kong A. W.-K. (2023). “TF-icon: diffusion-based training-free cross-domain image composition,” in Proceedings of the IEEE/CVF International Conference on Computer Vision (ICCV) (Paris: IEEE), 2294–2305. 10.1109/ICCV51070.2023.00218

[B16] Nusinovici S. Tham Y. C. Yan M. Y. C. Ting D. S. W. Li J. Sabanayagam C.. (2020). Logistic regression was as good as machine learning for predicting major chronic diseases. J. Clin. Epidemiol. 122, 56–69. 10.1016/j.jclinepi.2020.03.002 32169597

[B17] Oh S. Bang O. Y. Chung C.-S. Lee K. H. Chang W. H. Kim G.-M. (2012). Topographic location of acute pontine infarction is associated with the development of progressive motor deficits. Stroke 43, 708–713. 10.1161/STROKEAHA.111.632307 22343639

[B18] Pérez del Barrio A. Esteve Domínguez A. S. Menéndez Fernández-Miranda P. Sanz Bellón P. Rodríguez González D. Lloret Iglesias L.. (2023). A deep learning model for prognosis prediction after intracranial hemorrhage. J. Neuroimaging 33, 218–226. 10.1111/jon.13078 36585957

[B19] Pohl M. Hesszenberger D. Kapus K. Meszaros J. Feher A. Varadi I.. (2021). Ischemic stroke mimics: a comprehensive review. J. Clin. Neurosci. 93, 174–182. 10.1016/j.jocn.2021.09.025 34656244

[B20] Scarselli F. Gori M. Tsoi A. C. Hagenbuchner M. Monfardini G. (2008). The graph neural network model. IEEE Trans. Neural Netw. 20, 61–80. 10.1109/TNN.2008.2005605 19068426

[B21] Selvaraju R. R. Cogswell M. Das A. Vedantam R. Parikh D. Batra D. (2017). “Grad-cam: visual explanations from deep networks via gradient-based localization,” in Proceedings of the IEEE International Conference on Computer Vision (ICCV) (Venice: IEEE), 618–626. 10.1109/ICCV.2017.74

[B22] Seto H. Oyama A. Kitora S. Toki H. Yamamoto R. Kotoku J.. (2022). Gradient boosting decision tree becomes more reliable than logistic regression in predicting probability for diabetes with big data. Sci. Rep. 12:15889. 10.1038/s41598-022-20149-z 36220875 PMC9553945

[B23] Shan X. Li X. Ge R. Wu S. Elazab A. Zhu J.. (2023). “GCS-ichnet: assessment of intracerebral hemorrhage prognosis using self-attention with domain knowledge integration,” in IEEE International Conference on Bioinformatics and Biomedicine (BIBM) (IEEE: Istanbul, Turkiye), 2217–2222. 10.1109/BIBM58861.2023.10385726

[B24] Shen Y. Fang Z. Zhuang K. Zhou G. Yu X. Zhao Y.. (2025). Csf-net: Cross-modal spatiotemporal fusion network for pulmonary nodule malignancy predicting. arXiv preprint arXiv:2501.16400. 10.1109/ISBI60581.2025.10981213

[B25] Shimmyo K. Obayashi S. (2024). Fronto-cerebellar diaschisis and cognitive dysfunction after pontine stroke: a case series and systematic review. Biomedicines 12:623. 10.3390/biomedicines12030623 38540236 PMC10967718

[B26] Van der Maaten L. Hinton G. (2008). Visualizing data using T-SNE. J. Mach. Learn. Res. 9, 2579–2605. Available online at: https://jmlr.org/papers/v9/vandermaaten08a.html

[B27] Van Zandvoort M. De Haan E. Van Gijn J. Kappelle L. J. (2003). Cognitive functioning in patients with a small infarct in the brainstem. J. Int. Neuropsychol. Soc. 9, 490–494. 10.1017/S1355617703000146 12666773

[B28] Wang W. Xiao X. Liu M. Lan Q. Huang X. Tian Q.. (2024). “Multi-dimension transformer with attention-based filtering for medical image segmentation,” in IEEE 36th International Conference on Tools with Artificial Intelligence (ICTAI) (IEEE: Herndon, VA, USA), 632–639. 10.1109/ICTAI62512.2024.00095

[B29] Wang Y. Sun Y. Liu Z. Sarma S. E. Bronstein M. M. Solomon J. M. (2019). Dynamic graph cnn for learning on point clouds. ACM Trans. Graph. 38, 1–12. 10.1145/3326362

[B30] Wang Y. Wang C. Wei Y. Miao P. Liu J. Wu L.. (2022). Abnormal functional connectivities patterns of multidomain cognitive impairments in pontine stroke patients. Hum. Brain Mapp. 43, 4676–4688. 10.1002/hbm.25982 35770854 PMC9491282

[B31] Wu W. Qiu X. Song S. Chen Z. Huang X. Ma F.. (2024). Image augmentation agent for weakly supervised semantic segmentation. arXiv preprint arXiv:2412.20439. 10.1016/j.neucom.2025.131314

[B32] Xiao X. Zhang Y. Nguyen T.-H. Lam B.-T. Wang J. Hamm J.. (2025). Describe anything in medical images. arXiv preprint arXiv:2505.05804. 10.48550/arXiv.2505.05804

[B33] Xin Y. Luo S. Zhou H. Du J. Liu X. Fan Y.. (2024). Parameter-efficient fine-tuning for pre-trained vision models: a survey. arXiv preprint arXiv:2402.02242. 10.48550/arXiv.2402.02242

[B34] Xiong C. Chen H. Zheng H. Wei D. Zheng Y. Sung J. J.. (2024). “Mome: mixture of multimodal experts for cancer survival prediction,” in International Conference on Medical Image Computing and Computer-Assisted Intervention (MICCAI) (Springer: New York), 318–328. 10.1007/978-3-031-72083-3_30

[B35] Yan S. Xiong Y. Lin D. (2018). Spatial temporal graph convolutional networks for skeleton-based action recognition. Proc. AAAI Conf. Artif. Intell. 32, 7444–7452. 10.1609/aaai.v32i1.12328

[B36] Yang H. Liu H. Zhang K. Zong C. Wang A. Wang Y.. (2023). Neuroimaging markers of early neurological deterioration in acute isolated pontine infarction. Neurol. Sci. 44, 3607–3614. 10.1007/s10072-023-06837-2 37246178

[B37] Yang J. Awais M. Hossain M. A. Yee L. Haowei M. Mehedi I. M.. (2023). Thoughts of brain eeg signal-to-text conversion using weighted feature fusion-based multiscale dilated adaptive densenet with attention mechanism. Biomed. Signal Process. Control 86:105120. 10.1016/j.bspc.2023.105120

[B38] Yang J. Li L. Por L. Y. Bourouis S. Dhahbi S. Khan A. A. (2024). Harnessing multimodal data and deep learning for comprehensive gait analysis in pediatric cerebral palsy. IEEE Trans. Consum. Electron. 70, 5401–5410. 10.1109/TCE.2024.3482689

[B39] Yang J. Yang J. Yu X. Qiu P. Prajapat S. (2025). “D2-mlp: dynamic decomposed mlp mixer for medical image segmentation,” in ICASSP 2025-2025 IEEE International Conference on Acoustics, Speech and Signal Processing (ICASSP) (IEEE: Hyderabad, India), 1–5. 10.1109/ICASSP49660.2025.10888284

[B40] Yang L. Wu H. Jin X. Zheng P. Hu S. Xu X.. (2020). Study of cardiovascular disease prediction model based on random forest in eastern china. Sci. Rep. 10:5245. 10.1038/s41598-020-62133-5 32251324 PMC7090086

[B41] Yu X. Li X. Ge R. Wu S. Elazab A. Zhu J.. (2024). “ICHPRO: intracerebral hemorrhage prognosis classification via joint-attention fusion-based 3D cross-modal network,” in IEEE International Symposium on Biomedical Imaging (ISBI) (Athens: IEEE), 1–5. 10.1109/ISBI56570.2024.10635317

[B42] Zheng S.huai, Zhu, Z. Liu Z. Guo Z. Liu Y. Yang Y. Zhao Y. (2022). Multi-modal graph learning for disease prediction. IEEE Trans. Med. Imaging 41, 2207–2216. 10.1109/TMI.2022.3159264 35286257

[B43] Zhou H. Yu Y. Wang C. Zhang S. Gao Y. Pan J.. (2023). A transformer-based representation-learning model with unified processing of multimodal input for clinical diagnostics. Nat. Biomed. Eng. 7, 743–755. 10.1038/s41551-023-01045-x 37308585

[B44] Zong C. Liu H. Zhang K. Yang H. Wang A. Wang Y.. (2022). Prediction of symptoms on admission with early neurological deterioration in single small subcortical infarct. Curr. Neurovasc. Res. 19, 232–239. 10.2174/1567202619666220707094342 35796446

